# Supplementary figures and images for: Three-Year Intervention Effects on Food and Beverage Intake—Results from the Quasi-Experimental Copenhagen School Child Intervention Study (CoSCIS)
Source: Int J Environ Res Public Health. 2021 Oct 8;18(19):10543. doi: 10.3390/ijerph181910543 (PMC8507748; doi:10.3390/ijerph181910543)

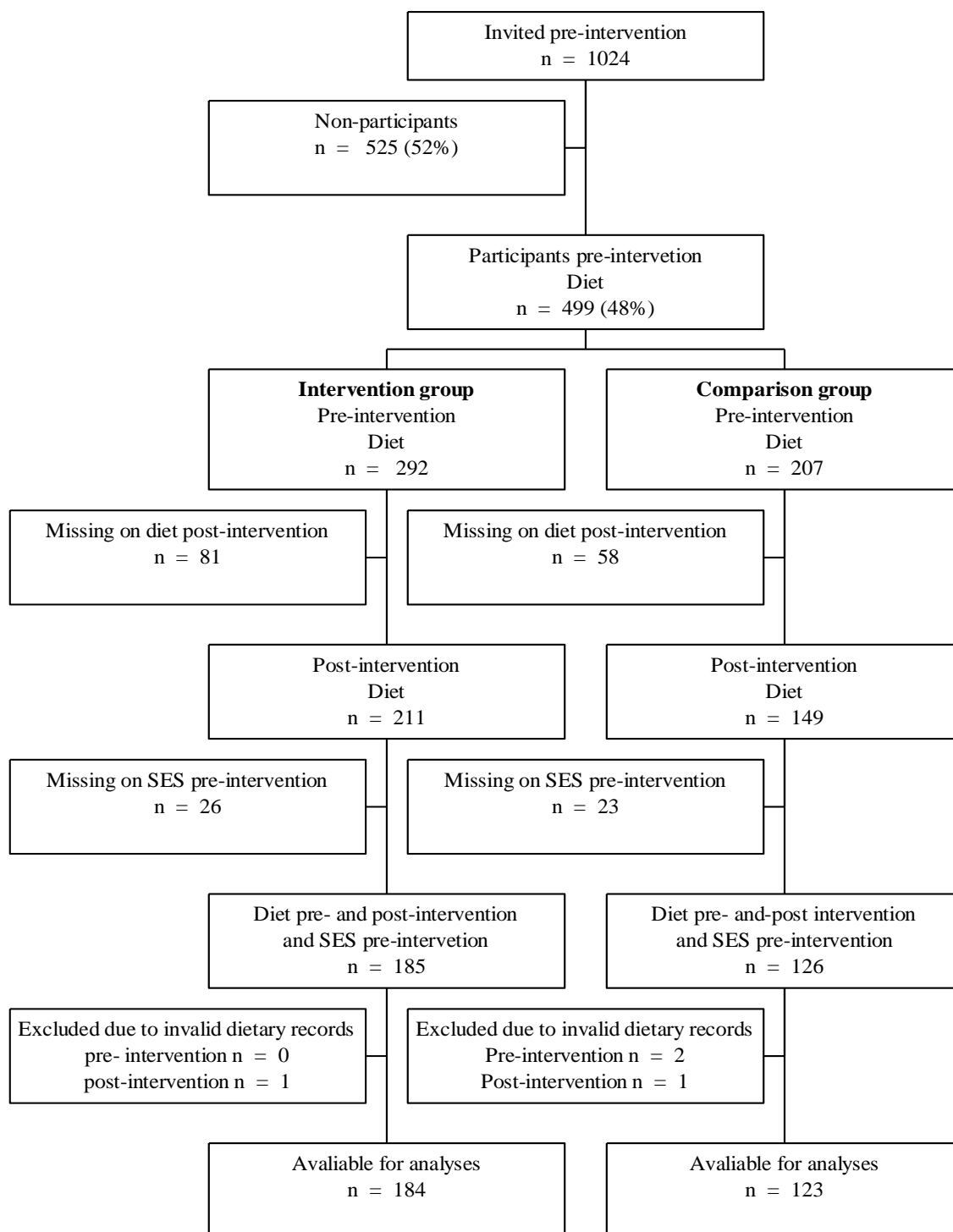

**Figure S1** Flow chart of the study population

Supplement: Supplementary file 1 [file ijerph-18-10543-s001.zip › ijerph-1373052-supplementary.pdf]
